# Study of Healthcare Professionals’ Interaction in the Patient Records Based on Annotations

**DOI:** 10.1007/978-3-030-51517-1_28

**Published:** 2020-05-31

**Authors:** Khalil Chehab, Anis Kalboussi, Ahmed Hadj Kacem

**Affiliations:** 8 Digital Research Centre of Sfax, Sfax, Tunisia; 9 Institut Mines-Télécom, CNRS, Paris, France; 10 Université de Sherbrooke, Sherbrooke, QC Canada; 11 Digital Research Centre of Sfax, Sfax, Tunisia; 12 University of Sfax, Sfax, Tunisia; 13 ReDCAD Research Laboratory, Faculty of Economics and Management, University of Sfax, Sfax, Tunisia; 14 Higher Institute of Computer Science and Management, University of Kairouan, Kairouan, Tunisia; 15 ReDCAD Research Laboratory, University of Sfax, Sfax, Tunisia

**Keywords:** E-heath, Annotation, Classification, Health record, Annotation system, Healthcare professionals

## Abstract

The annotation practice is an almost daily activity; it is used by healthcare professionals (PHC) to analyze, collaborate, share knowledge and communicate, between them, information present in the healthcare record of patients. These annotations are created in a healthcare cycle that consists of: diagnosis, treatment, advice, follow-up and observation.

Due to an exponential increase in the number of medical annotation systems that are used by different categories of health professionals, we are faced with a problem of lack of organization of medical annotation systems developed on the basis of formal criteria. As a result, we have a fragmented image of these annotations tools which make the mission of choice of an annotation system by a PHC, in a well-defined context (biology, radiology…) and according to their needs to the functionalities offered by these tools, are difficult.

In this article we present a classification of thirty annotation tools developed by industry and academia based on 5 generic criteria. We conclude this survey paper with model proposition.

## Introduction

The paper annotation practice is very common. Indeed, during our reading we are all accustomed to scribble our comments in a margin of document, to highlight, to circle sections, to paste post-it…, which aims to enrich and add value to information [40, 43]. Annotation is a central practice in many professions: teachers annotate copies of students; professors exchange annotated documents during their work; Engineers co build engines by annotating sketches of plans to make them evolve, doctors comment on patient folder, etc. [39, 44].

Annotations thus, take various forms and are used for different functions [28]. Moreover, computerization of documents offers us new perspectives to use these annotations (indexation, creation, document, assistance, etc.) which do not exist on paper [41]. Assistance is a very important function that can be related to the annotation activity.

Annotative activity is different when dealing with the professional annotator case. These annotations are created in a specific context, will follow a path, developed for the purpose of determining a specific task etc.

The annotation is expanded in a document flow. The latter is a carrier of the ratings. These annotations processed in this context are complementary. They provide us with information scattered over different documents. In fact, if we try to understand each one separately, in other words; if we separate an annotation from its creative context, we find that it does not make sense. In short, the annotation can be understood only in its semantic field.

For this reason, the study of annotation in a professional context obliges us to implement an annotation model that describes what actually happens. This model must provide us with the necessary links either at the level of the annotated documents or at the level of the tasks made without forgetting to take into account the specificity of the annotation’s production domain. The documents that usually belong to a folder that is made for the purpose of carrying out a task has a definite circuit that repeats itself each time one needs to do this task. The proper understanding of annotations can be done in its creative context.

Doctors in/after consultation [45] use internet to search information that can help him. Based on their annotations it’s possible to assist doctors and to gives him automatically pertinent information, from the net, after studying and analyzing their annotations.

This paper is organized as follows: Sect. 2 gives a classification of these tools based on several criteria; Sect. 3 gives a model proposition. Finally, Sect. 4 concludes this article.

## Health Record Annotations and Medical Annotation Systems

In this work, we started with an exhaustive reading for the available papers on medical annotation systems (academic annotation system) and viewing the existing industrial annotation systems in the e-health domain. Although the medical annotation systems have already been studied in a variety of contexts, yet when it comes to the PHC to choose which system to use it is not a trivial task neither for a researcher to identify future research areas. This is because the annotation systems are so common and many of them share similar objectives. Moreover, there are no formal criteria to facilitate the comparison between those systems and to guide PHC choice or a researcher. As a result, there is a fragmented picture of these annotation tools. As far as we know, this is the first work to consider the classification of medical annotation system. When we determined the study of these systems we deduced that there are several common criteria which can classify these later. Several studies have proposed classifications of annotation systems in several fields [34, 35, 37] or in the field of e-health [42, 43]. We propose a classification based on 5 criteria which are: type of medical annotation object, the medical annotation activity, healthcare professional (Practitioner), type of annotation system, type of annotated resource.

### Type of Medical Annotation Object (Cognitive/Computational)

- **Cognitive:** this annotation is created to be used by a human agent. In this case, the annotation requires a cognitive and intellectual effort to be interpreted. This annotation has a visible visual form on the document [34].
- **Computational:** this annotation is intended to be processed and manipulated by software agents. These annotations are also called meta-data. They allow us to annotate computer resources to facilitate their exploitation by machines.

### The Medical Annotation Activity (Manual, Automatic, Semi-automatic)

Annotation activity begins with the choice of anchor and annotation form in the annotation toolbar related to the annotation software. Then, the annotation must complete the properties of the annotation; this process ends with the attachment of the annotation to a well-defined target. Based on this process we can classify the annotative activity as: manual, semi-automatic or automatic [38].

- **Manual:** the process already mentioned will be carried out totally by the user himself, who selects the form of the annotation, selects the anchor and creates the annotation. This process is similar to the process of annotation when a paper support is available.
- **Automatic**: the annotation process already mentioned is carried out totally by the machine. These annotations are based on either context sensors or pattern recognition techniques, etc.
- **Semi-automatic:** in this case, the process will be done from the start by the user. After a while, the system acquires and understands the way the user annotates. It moves to a suggestion of annotations that are automated, based on an annotation model built with rules under development. At this stage, human intervention remains just to validate or not validate and to refine the annotation rules created at a certain level, where there are no corrections and there is complete acceptance of the suggested rules, human intervention is canceled and the process becomes totally automated.

### Healthcare Professional (Practitioner)

It is the annotator that is equipped with an annotation system to use all the functionalities offered by the latter. In our case, the practitioners are healthcare professionals (doctor, nurse, biologist, and radiologist). The healthcare cycle is composed of four phases (diagnostic, treatment, advice, follow up and observation). Each practitioner, with a medical annotation system, intervenes in one or many phases, according to their role, to accomplish a specific task in which annotation is made.

### Type of Annotation System

- **Application:** an application is created to annotate the resources already consulted. These applications offer several functionalities as the types below.
- **Plug-in:** these are the expansion modules, an external module that is added to a website or software and which will make it possible to provide annotation functionalities to the latter.
- **Website:** these are specialized websites to annotate consulted resources by registered users on the web.

Knowing the type of annotation system facilitates the development of a patient record model which will be proposed in future research. This model allows communicating with different types of medicals annotations systems.

### Type of Annotated Resource

Annotated resources can be: word document, pdf, image, text, video, html, audio, etc.

Table 1 presents a comparative study of the medical annotation systems seen in the bibliographical study using the 5 criteria already explained.

**Table 1.** Comparative study of the medical annotation systems using 5 criteria

Name of annotation system		Year	Healthcare professional (Practitioner)	Annotated resource type	Category of annotation system			Annotation type		Type of annotation activity		
					Application	Plug-in	Web	Cognitive	Computational	Manual	Automatic	Semi-automatic
Santo	[1]	2018	D	Text			×	×			×	
Clean tools	[2]	2018	D	Image 3D	×			×		×		
Epivizr	[3]	2018	B	Document	×			×		×	×	
ODMSummary	[4]	2017	D	HTML			×	×			×	
3dBionote	[5]	2017	B	Image			×		×	×		
Verdant	[6]	2017	B	Text			×		×		×	
Best slice	[7]	2017	R, D	Image			×	×		×		
MicroMD	[8]	2017	R, D	Image	×			×		×		
GIDAC	[9]	2016	R	Image	×			×		×		
Vcf-miner	[10]	2016	B	Text			×		×	×		
Plexo	[11]	2016	R	Image		×		×		×		
BioDigital human	[12]	2016	D	Image 3D	×			×		×		
Icare	[13]	2015	N	Document	×			×		×		
Heideltime	[14]	2015	A	Time	×			×		×		
Domeo Annotation	[15]	2014	B	HTML		×			×	×	×	×
BioR	[16]	2014	B	Text		×			×	×	×	
3dmarkup radiologist	[17]	2014	R	Image			×		×	×		
Vita	[18]	2014	R	Image, video	×			×		×		
Marky	[19]	2014	R	All type	×				×			×
Cliosoft dental	[20]	2014	D	Image			×	×		×		
Medetect	[21]	2013	D	HTML	×				×			×
Flersa	[22]	2012	R	Image	×			×		×		×
SMItag	[23]	2012	R	Image			×		×	×		
Mammoapplet	[24]	2012	R	Image			×	×		×		
Brat	[25]	2012	A	Text	×			×		×		
Idash	[26]	2012	D	Text	×			×		×		
MedAt	[27]	2011	D	Document	×				×			×
@note	[28]	2009	B	text	×				×		×	
Arthemis	[29]	2007	R	Video	×			×		×		
DocAnnot	[30]	2006	D, N	Document	×							×

B: Biologist, D: Doctor, N: Nurse, R: Radiologist, A: all healthcares professional

Table 1 presents a comparative study of thirty medical annotation systems seen in the bibliographical study using the 5 criteria already explained. These annotation systems are ranged on the table according of the chronological order of their publication year.

## Model Proposition

Several models are already seen in the literature [33, 35, 36] and [ 37], these models present many problems, like the inexistence of the modeling of the cycle of care, the consideration of the annotation as an objective, no invocation of the services linked to the annotation, that not allows to use it in the healthcare domain. For this reason and based on the classifications already seen (Sect. 2) we propose an annotation model that preserve the semantic of annotative activity in the health domain.

### Concept of Model

Our model must reflect the annotation process actually done; it must contain the following concepts:

- Basic_Concept: This group contains the concepts that can exist in each domain.
  - Place: this is the physical place where the annotation is produced by the annotator.
  - Anchor: this is the position of the annotation on the document.
  - Shape: represents the visual aspect of the annotation.
  - Annotated_content: this is the annotated passage.
  - Annotating_content: this is the comment written by the annotator about the Annotated_content.
  - Type: the annotated content can have a type that enhances its content and facilitates access, filtering, searching later.
  - Device: it is the device used to read and annotate the document.
  - Date: represents the creation date of the annotation.
  - Name: the name of PHC.
- Department: it is a part of decomposing the tasks of an organization (Hospital) according to the functions or the nature of these activities. This appointment is used in the professional field.
  - Service: It is a part of decomposing the tasks of a department according to the functions or the type of these activities.
  - Professional (PHC): it is the person (professional healthcare) who reads a document and makes the annotations.
  - Role: it is the function occupied by a professional.
  - Authority: it’s the set of tasks that can be done by a professional in his role.
  - Name: it’s the name and surname of the annotator.
- Package_document: it’s a grouped document set.
  - Document: it’s the annotated document.
  - Part_document: professional documents are usually divided into parts.
  - Element: each Part_document consists of a set of elements.
  - Part_element: it is the smallest granularity of document; it is a word, letter etc. In short, it is the annotated passage.
- Specific_concept: in each domain where the annotators are professionals, there are specific concepts related to the latter.
  - Validity: annotation can be associated with date which contains: Start_date, Finish_date, and Cyclic_date. Example: control the vital parameters of the patient today from 8 h (Start_date) to 19 h (Finish_date) every 2 h (Cyclic_date).
  - Scope: specifies the professional that can view the annotation.
  - Importance: a value affected to the annotation which valorizes it.
- Medical_care: each healthcare cycle consists of a set of Healthcare_cycle. Each Healthcare_cycle consists of a:
  - Diagnostic: Contain a detailed anamnesis of:
    - Patient history
    - Family history
    - Socio-economic question
    - Symptom of illness
    - Date of illness
    - Treatment already followed
    - Physical examination of patient
    - Complementary examination: medical image, biological analysis….
- Treatment: it contains the treatment written by the doctor.
- Advice: patient education: how to live with this disease, how to act in case of emergency, how to take their drugs…
- Follow up and observation: this is the last step of the care cycle in which the doctor follows his patient until the stabilization of his condition. In this step, it can also make a strengthening of advice….
- Service aspect: this aspect allows us to clarify the semantic of the annotation. It is used to interpret the meaning of the annotation by the annotator himself or by the software agents.
  - Effect: the effect of annotation is the result of web service called from this annotation.

### Relationships Between Concepts

**Fig. 1.**
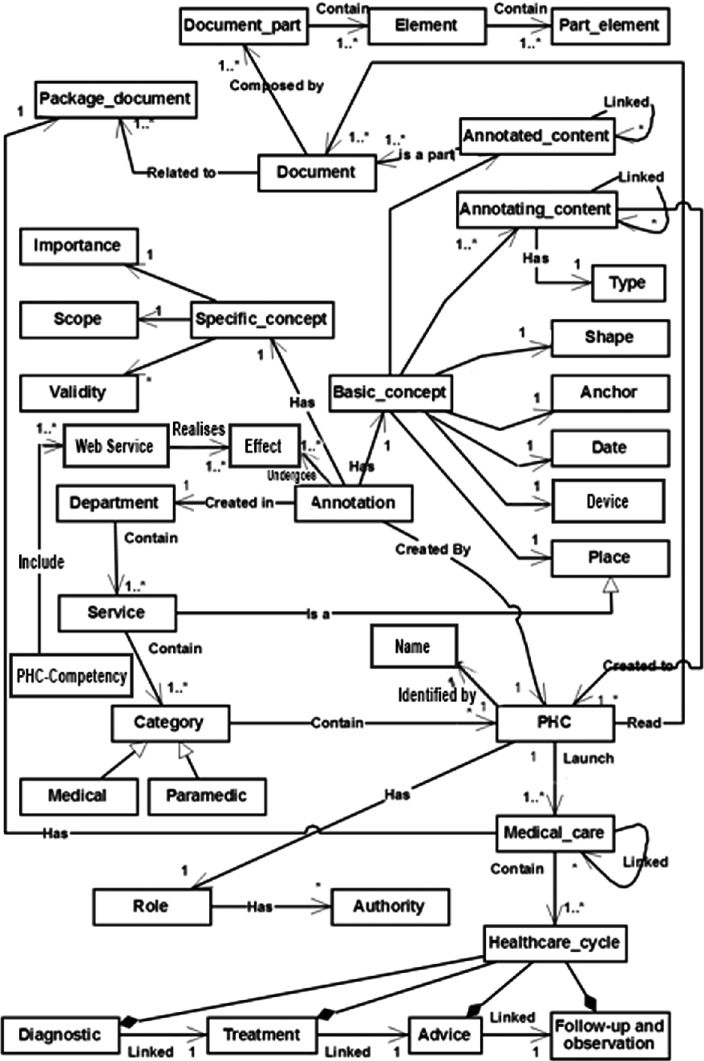
PHC annotation model

The relationships between the concepts of ontology are described through the data model presented in Fig. 1 as follows. There are only three type of cardinalities used in this model 1..*, 1 and *. An annotation is created by the PHC with a device, at a date and in a place. This annotation is presented by a particular shape. It is pointed to an anchor and related to an annotated content that can be related to another annotated content. This annotated content is a part of the read document. The annotation contains an annotating content that can be related to others annotating contents. An annotating content can have a type. An annotation has an annotated content that is alone, and at the base of this content, annotating content can be created for one or more professionals since this content can be written differently for each destination. Each domain has some specific concept. An annotation is related to Specifics concept.

The annotator, which is a PHC, is identified by a name. The place is a location in a medical department especially in a specific service. Each service has its own PHCs that are classified into categories (medical, paramedic). In the category a PHC can have a role and each role is related to a specific authority. A document is an element of a document package. A document package consists of the already known set of documents grouped together to perform a specific task. A document decomposed to parts, each one of it contains a set of elements (text box, radio button…). The annotated content can be an element or a part of an element (word, sentence…). A professional can have a power to launch tasks (Medical_care). Each healthcare cycle is divided into healthcare phases (Diagnostic – Treatment – Advice - Follow up and observation); the latter are linked together so we can fuse them once again when they are completed and we can follow the annotations of a healthcare cycle at the level of its phase. Each healthcare phase can be divided itself. A healthcare cycle can also be linked to other healthcare cycle that has a relationship between them.

## Conclusion and Future Work

In this paper, we studied annotation systems of the digital health domain available in industrial and research areas in order to propose a unified classification of this kind of system that is omnipresent in hospital information systems. This panoramic view provided is based on the classification of thirty different annotation systems developed in the literature over the past two decades. This organization of annotation tools is built on the basis of five criteria: type of annotation (computational/cognitive); category of annotation system (application/plug-in/website); type of annotative activity (manual/semi-automatic/automatic); type of annotated resource (text/Web page/video/image/database) and practitioner (biologist/doctor/radiologist/nurse, etc.). This classification based on criteria, already explained in our study, which are transversal organizational criteria, facilitates the identification of limitations and possible challenges in the area of the medical annotation systems. Based on this, we proposed an ontology that covers the identified challenges and lead to a more intelligent annotation system. In future research, we try to use the results of this study to create an annotation template for PHCs and then try to generalize them to be functional for all professionals in different domains. We are also trying to create the computer services which allow the PHC to be assisted throughout the care cycle.
